# Efficacy and safety of sofosbuvir in the treatment of hep C among patients on hemodialysis: a systematic review and meta-analysis

**DOI:** 10.1038/s41598-020-71205-5

**Published:** 2020-08-31

**Authors:** Fadi Shehadeh, Markos Kalligeros, Katrina Byrd, Douglas Shemin, Eleftherios Mylonakis, Paul Martin, Erika M. C. D’Agata

**Affiliations:** 1 Infectious Diseases Division, Warren Alpert Medical School of Brown University, Rhode Island Hospital, 593 Eddy Street, POB 328, Providence, RI USA; 2 Kidney Disease Division, Warren Alpert Medical School of Brown University, Rhode Island Hospital, Providence, RI USA; 3 Division of Digestive Health and Liver Disease, Miller School of Medicine, University of Miami, Miami, FL USA

**Keywords:** Hepatology, Infectious diseases

## Abstract

Hepatitis C virus (HCV) infection among maintenance hemodialysis patients is implicated in increased morbidity and mortality compared to uninfected patients. Sofosbuvir (SOF)-based regimens may not be optimal among patients requiring hemodialysis. Several studies, however, provide evidence that use of SOF among HCV-positive patients with renal impairment, is effective and safe. We searched Pubmed and Embase to identify studies reporting the efficacy and safety of SOF-based regimens for the treatment of HCV-positive patients on maintenance hemodialysis and performed a random effects meta-analysis. The overall pooled estimate of the efficacy of SOF-based therapy was 95% (95% CI 91–98%). The efficacy of the SOF-based regimen was 92% (95% CI 80–99%), 98% (95% CI 96–100%), and 100% (95% CI 95–100%) for the following doses: 400 mg on alternate days, 400 mg daily, and 200 mg daily, respectively. The most frequent adverse event was fatigue with a pooled prevalence of 16% (95% CI 5–29%), followed by anemia 15% (95% CI 3–31%), and nausea or vomiting 14% (95% CI 4–27%). Anemia was more prevalent in treatment regimens containing ribavirin (46%, 95% CI 33–59%) compared to ribavirin-free regimens (3%, 95% CI 0–9%). This study suggests that SOF-based regimens in the treatment of HCV infection among hemodialysis patients are both effective and safe.

## Introduction

Patients requiring hemodialysis are at an increased risk of acquiring hepatitis C virus (HCV)^1^. Approximately 5% of patients on hemodialysis are diagnosed with HCV, compared to 1% in the United States population^2,3^ and, among hemodialysis patients, HCV infection is associated with poor outcomes with substantially higher rates of hepatic-related mortality and hospitalizations, and lower health-related quality of life scores, compared to HCV negative hemodialysis patients^4^.

National guidelines for the treatment of HCV infection among hemodialysis patients recommend several direct-acting antivirals (DAA)^5,6^. Sofosbuvir (SOF)-based regimens are not recommended for patients requiring hemodialysis since renal clearance of SOF is the major elimination pathway, although since the recent U.S. Federal Drug Administration change in label, removing restrictions on its use in renal impairment, this will undoubtedly change^7^. Nevertheless, clinical studies provide evidence that use of SOF, among HCV positive patients with renal impairment, is effective and safe^8,9^. Data focusing only on patients requiring maintenance hemodialysis are limited. Thus, we performed a systematic review and meta-analysis to quantify the efficacy and safety of SOF-based regimens among the subset of patients with severe renal impairment that required maintenance hemodialysis.

## Results

**Figure 1:**
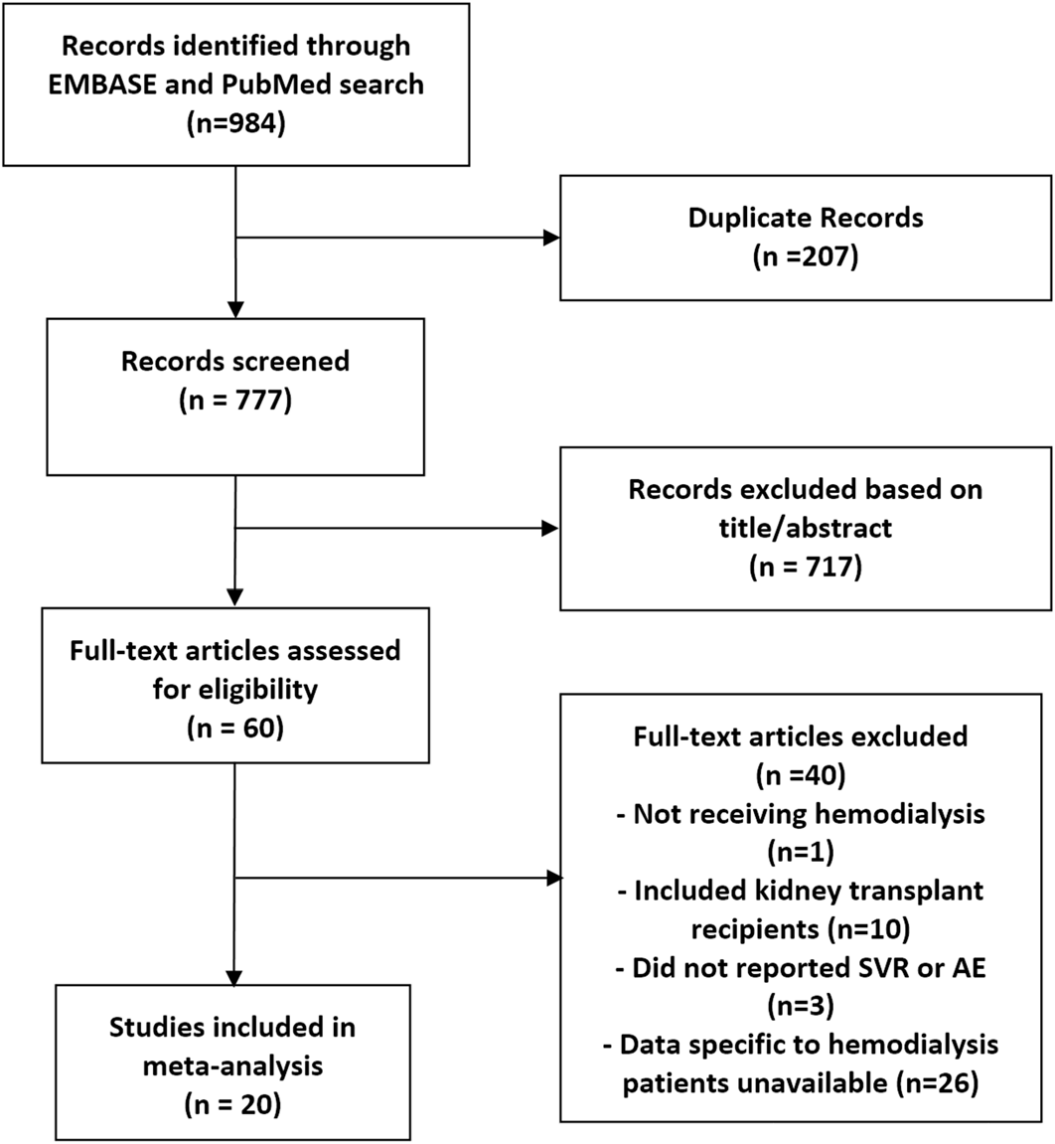
Flow diagram for selection of studies included in the systematic review and meta-analysis. *SVR* sustained virologic response, *AE* adverse events.

Our systematic search yielded 777 studies of which 60 were eligible for full text review. A total of 20 studies were in accordance with our eligibility criteria and were included in the meta-analysis (Table 1). The selection process is detailed in Fig. 1.

**Table 1:** Summary of study and patient characteristics.

Study	Study period	Type of study	Country	Population (N)	Age (mean)	Male (%)	Genotype (%)	Combination drugs	Sofosbuvir dose	SVR12 N (%)
Agarwal et al*.*^14^	2015–2016	Prospective	India	62	33.8	66.1	GT-1: 65, GT-2: 2, GT-3: 29, GT-4: 3, GT-6: 2	RBV, DCV	400 mg AD, 400 mg daily	59 (95%)
Akhil et al*.*^10^	2015–2016	Retrospective	India	22	49.76	68.2	GT-1: 64, GT-3: 27, GT-4: 9	RBV, DCV	400 mg daily	16 (73%)
Bhamidimarri et al*.*^15^	2014–2015	Prospective	USA	10	59.7	91.7	GT-1a: 67, GT-1b: 33	SMV	200 mg daily, 400 mg AD	10 (83%)
Choudhary et al*.*^16^	2015–2016	Prospective	India	2	56.5	100	GT-1: 50, GT-3: 50,	DCV	400 mg AD	2 (100%)
Desnoyer et al*.*^17^	2014–2015	Prospective	France	12	54.4	83.3	GT-1: 92, GT-2: 8	SMV, DCV, LDV, RBV	400 mg daily, 400 mg 3 times/week	10 (83%)
Gupta et al*.*^18^	2015–2016	Prospective	India	7	48.4	71.4	GT-1: 57, GT-3: 43	DCV, RBV	200 mg daily	6 (86%)
He et al*.*^19^	2016	Prospective	China	33	52.8	72.7	GT-1a: 21, GT-2a: 73, GT-1b + 2a:6	DCV	200 mg daily	33 (100%)
Mehta et al*.*^20^	2016	Prospective	India	38	49.5	68.4	GT-1a: 42, GT-1b: 58	LDV, DCV	400 mg daily, 400 mg AD	33 (87%)
Sperl et al*.*^11^	2015–2016	Retrospective	Czech Republic	6	39	100	GT-3:100	DCV	200 mg daily	6 (100%)
Surendra et al*.*^22^	2015	Prospective	India	21	44	61.9	GT-1a: 57, GT-1b: 33	LDV	400 mg AD	19 (90%)
Singh et al*.*^21^	2014–2015	Prospective	USA	8	56.8	25	GT-1a: 38, GT-1b: 38, GT-3: 13, GT-4:13	SMV, LDV	400 mg daily	7 (88%)
Borgia et al*.*^23^	2017–2018	Prospective	Canada, UK, Spain, Israel, New Zealand, Australia	59	60	35	GT-1a: 25, GT-1b: 19, GT-2: 12, GT-3: 32, GT-4: 7, GT-6: 3	VPV	400 mg daily	56(95%)
Seo et al*.*^13^	2017–2018	Retrospective	Korea	9	59.9	66.7	GT-2:100	RBV	400 mg daily	9 (100%)
Lin et al*.*^27^	2017	Prospective	China	7	53	71.4	GT-1b: 57, GT-2a: 29, GT-6: 14	DCV, LDV	400 mg daily	6 (86%)
Debnath et al*.*^25^	2017–2018	Prospective	India	18	39.4	77.8%	GT-1: 66.7, GT-2: 5.5, GT-3: 22.3, GT-5: 5.5	DCV, LDV	400 mg daily	18 (100%)
Singh et al*.*^29^	2015–2017	Prospective	India	39	39.6	83%	GT-1: 68, GT-3: 28, GT-4: 4.3	DCV, LDV	400 mg daily	37 (95%)
Mandhwani et al*.*^28^	2016–2018	Prospective	Pakistan	73	31.9	72.9%	GT-1: 50.3, GT-2: 0.7, GT-3: 42.9, GT-4: 1.48	DCV, RBV	400 mg daily	70 (96%)
Hussein et al*.*^26^	2017	Prospective	Iraq	19	54.8	63%	GT-1a:100	DCV, LDV	200 mg daily, 400 mg daily	19 (100%)
Gaur et al*.*^12^	2017–2018	Retrospective	India	31	39.8	77.5%	GT-1: 67.7, GT-3: 32.2	VPV	400 mg daily	30 (97%)
Cheema et al*.*^24^	2017–2018	Prospective	Pakistan	36	47.22 Group 1 53.89 Group 2	61.1%	GT-1: 33.3, GT-2: 2.8, GT-3: 63.9	DCV	400 mg daily (Group 1), 400 mg 3 times/week (Group 2)	29 (80%)

*NR* Not reported, *GT* genotype, *SVR12* sustained virologic response 12 weeks after treatment, *AD* alternate day, *RBV* ribavirin, *SMV* simeprevir, *LDV* ledipasvir, *DCV* daclatasvir, *VPV* velpatasvir.

The included studies were published from 2015 to 2020 and contained extractable data on 514 patients enrolled from 2014 to 2016. Four studies were retrospective^10–13^ and 16 were prospective^14–29^. Nine of the studies were conducted in India^10,12,14,16,18,20,22,25,29^, 2 in the USA^15,21^, 2 in Pakistan^24,28^, 2 in China^19,27^, 1 in France^17^, the Czech Republic^11^, Korea^13^, Iraq^26^, and 1 enrolled patients from multiple countries^23^. All studies received more than five stars in the Newcastle–Ottawa Scale and no study was excluded due to quality concerns.

**Figure 2:**
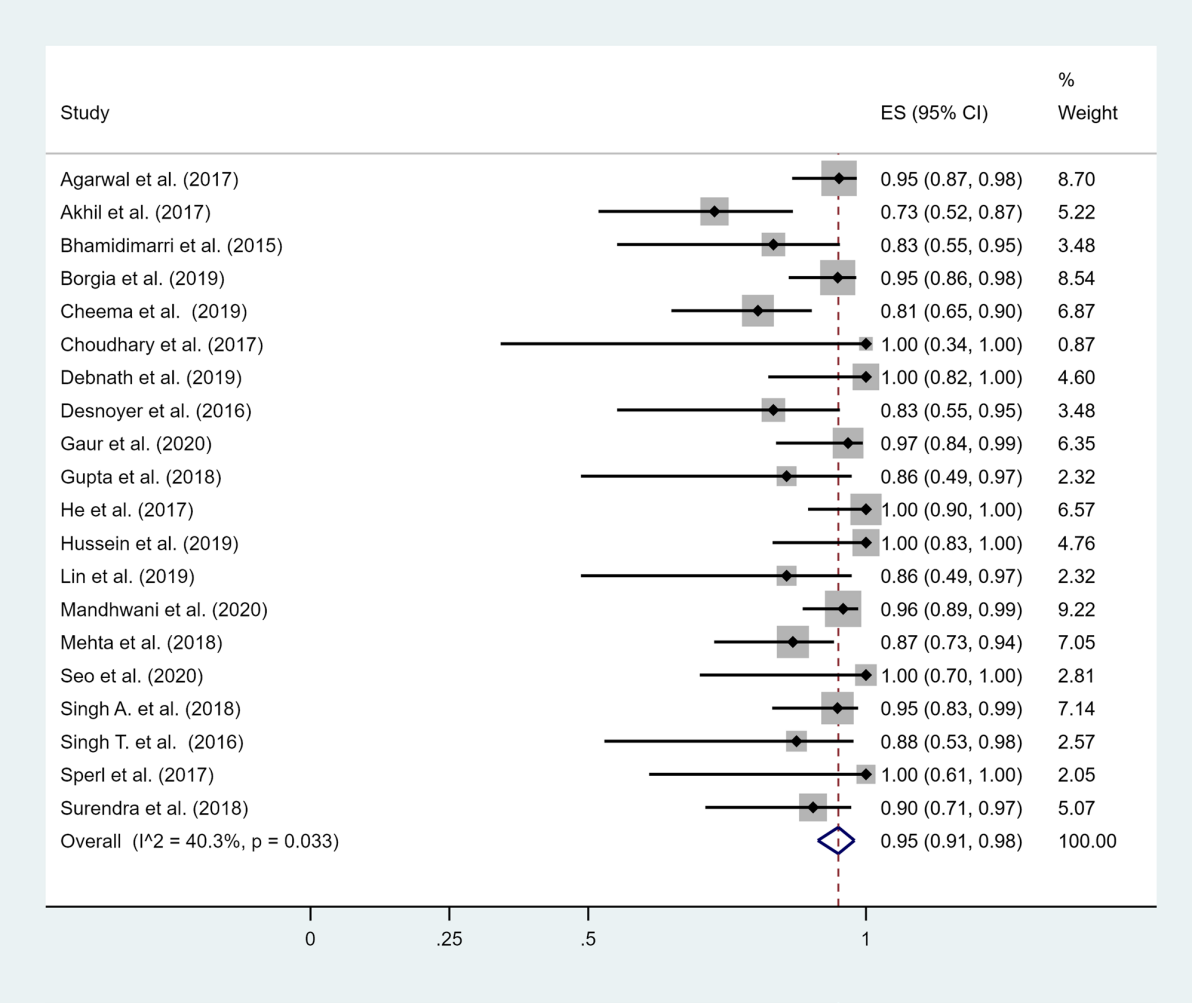
Forest plot of included studies. Individual and combined estimates of the efficacy of sofosbuvir-based therapy with 95% confidence intervals. *ES* effect size (efficacy).

The overall pooled estimate of the efficacy of SOF-based therapy among HCV positive patients on maintenance hemodialysis was 95% (95% CI 91–98%), ranging from 73 to 100% (Fig. 2). There was moderate heterogeneity among studies (I^2^ = 40.3%, p < 0.05). A meta-regression analysis did not demonstrate any evidence that the differences in gender, age, HCV genotypes, or type of SOF-based regimens among studies were correlated with the between study heterogeneity (p = 0.97). Egger’s test for publication bias yielded insignificant results (bias = − 0.76, p = 0.46), suggesting absence of small-study effects.

A total of 19 studies reported extractable data on the SOF dose^10–14,16–29^ and the other DAA included in the treatment regimen. In nine studies^10–13,16,19,21–23^, the same treatment regimen was used for all study participants. Six studies partitioned the study participants into subgroups and followed a different treatment strategy for each subgroup^14,17,18,20,24,25^. Each treatment strategy was considered as a separate observation in our analysis and was included separately in its respective subgroup.

**Figure 3:**
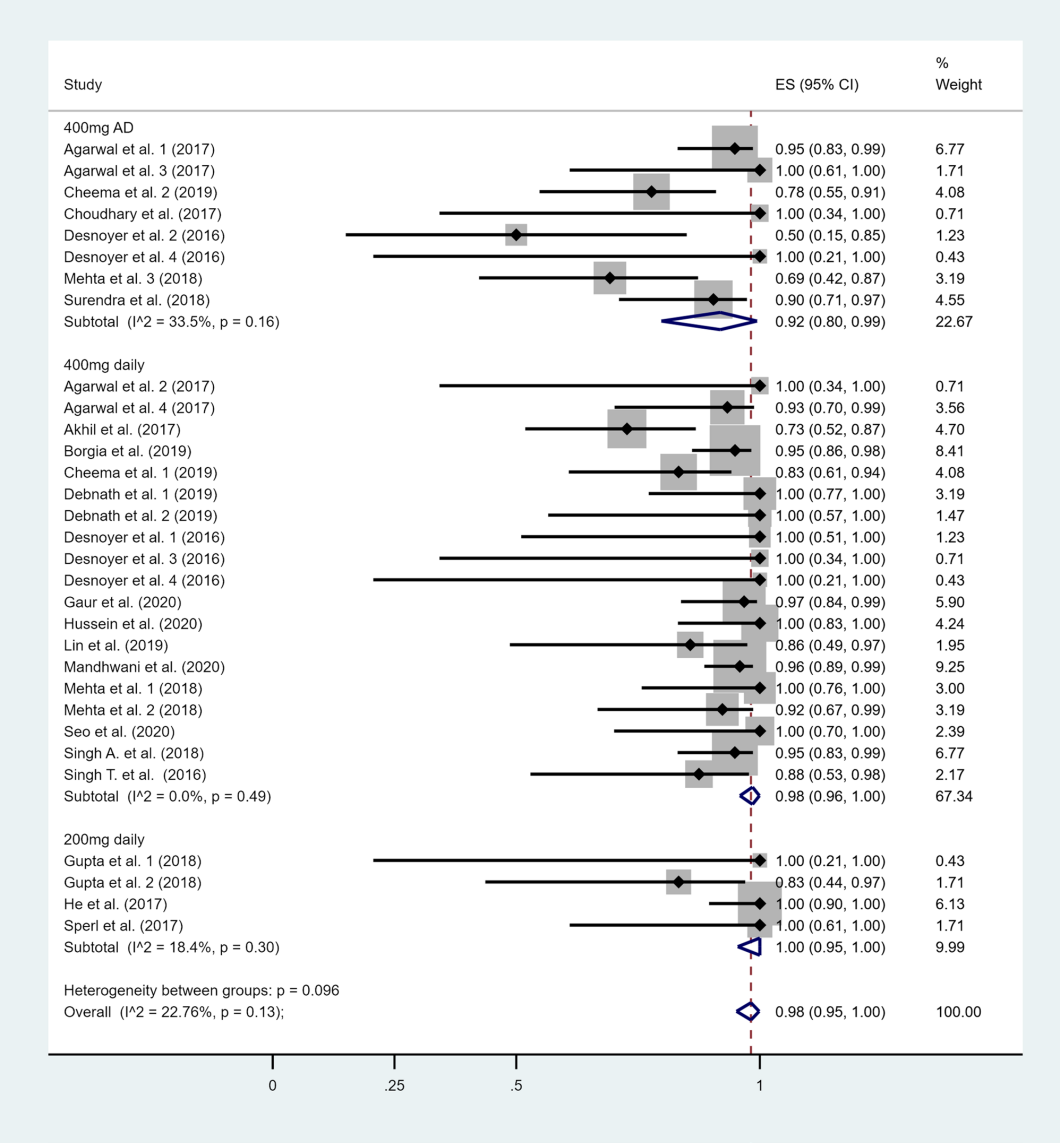
Forest plot of included studies sub-grouped by sofosbuvir dose. Individual and combined estimates of the efficacy of SOF-based therapy for each sub-group with 95% confidence intervals. *ES* effect size (efficacy).

The efficacy from the studies that reported extractable data on the treatment regimen used varied from 50 to 100% (Fig. 3). Studies were sub-grouped based on the SOF-dose administered. The pooled efficacy of the sofosbuvir regimen was 92% (95% CI 80–99%) in patients administered a 400 mg alternate day dose, 98% (95% CI 96–100%) in those administered a 400 mg daily dose, and 100% (95% CI 95–100%) in patients administered a 200 mg daily dose (Fig. 3). A sub-group analysis was not performed for the various other DAA used in the treatment regimens, due to the large variability of DAA used. Ribavirin was used in 6 studies^10,13,14,16,18,28^, simeprevir in 3^15,17,21^, ledipasvir in 8^17,20–22,25–27,29^, daclatasvir in 9^10,11,14,16–20,24–29^ and velpatasvir in 2 studies^12,23^ (Table 1).

Six studies included extractable data on the efficacy of SOF-based therapy in patients with cirrhosis^11,12,17–19,24^. The pooled estimate of the efficacy of SOF-based therapy among HCV positive patients on maintenance hemodialysis with cirrhosis was 97% (95% CI 84–100%), ranging from 67 to 100% (Supplementary Fig. 1).

**Figure 4:**
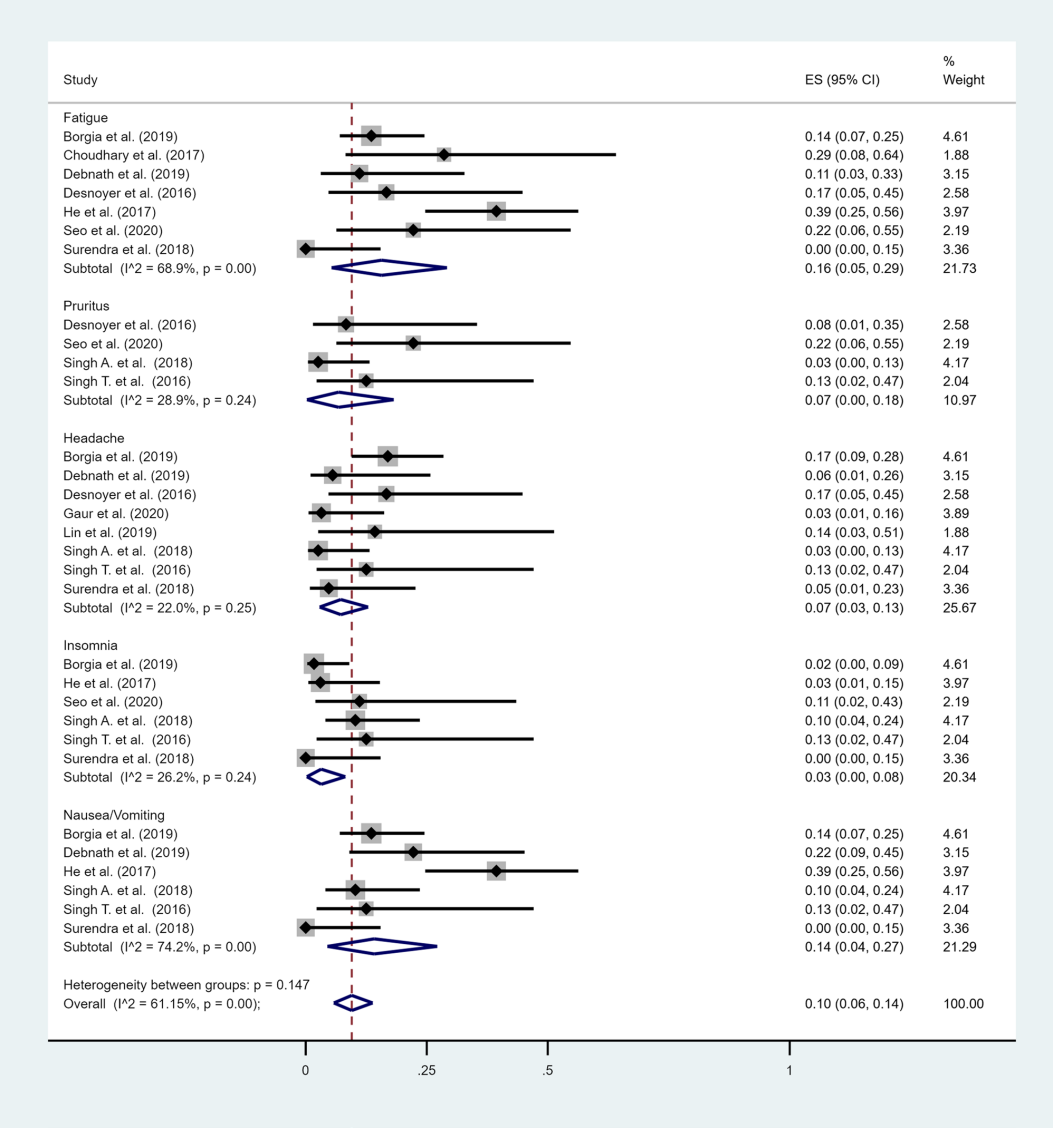
Forest plot of included studies that reported adverse events. Individual and combined estimates of prevalence of each adverse event with 95% confidence intervals. *ES* effect size (prevalence).

**Figure 5:**
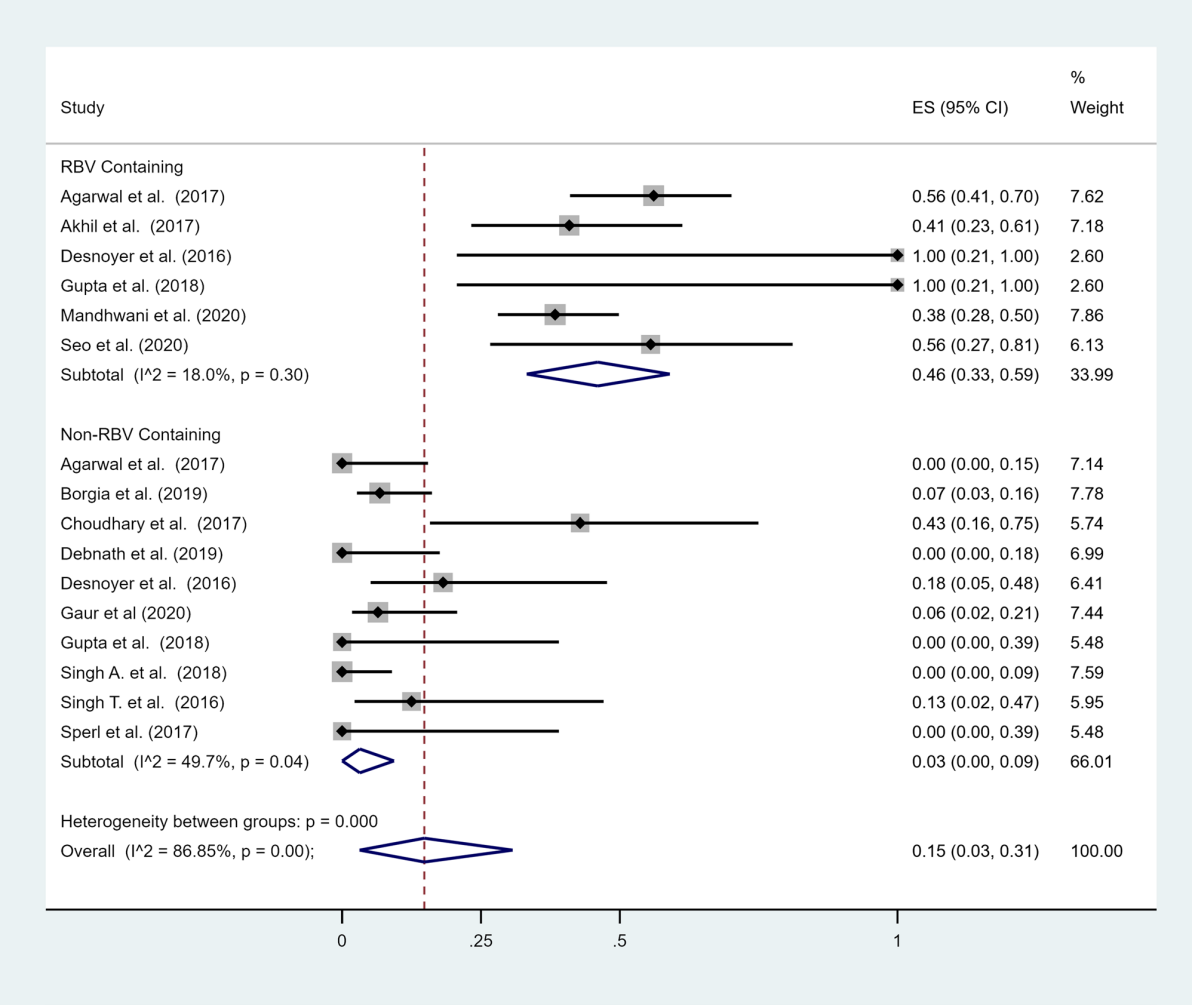
Forest plot of included studies that reported anemia as an adverse event. Individual and combined estimates of prevalence of each adverse event with 95% confidence intervals. *ES* effect size (prevalence).

A total of 16 studies reported extractable data on adverse events^10–14,16–19,21–23,25,27–29^ (Table 2). The most commonly reported adverse events were anemia (10 studies)^10,12–14,16–18,21,23,28^, headache (8 studies)^12,17,21–23,25,27,29^, fatigue (6 studies)^13,16,17,19,23,25^, nausea or vomiting (5 studies)^19,21,23,25,29^, insomnia (5 studies)^13,19,21,23,29^ and rash or itching (4 studies)^13,17,21,29^. The most frequent adverse event was fatigue with an overall pooled prevalence of 16% (95% CI 5–29%) (Fig. 4). Anemia ranged from 0 to 56%, with an overall pooled prevalence of 15% (95% CI 3–31%) (Fig. 5). Anemia was more prevalent in treatment regimens containing ribavirin (46%, 95% CI 33–59%) compared to regimens that did not contain ribavirin (3%, 95% CI 0–9%). The overall pooled prevalence of headache was 7% (95% CI 3–13%), nausea or vomiting 14% (95% CI 4–27%), insomnia 3% (95% CI 0–8%), rash or itching 7% (95% CI 0–18%). Two studies reported drug discontinuation due to adverse events^18,24^. Cheema et al. reported a treatment related serious adverse event (drug induced rash) in 1 patient^24^, and Gupta et al. reported that 1 patient developed recurrent hypoglycemia which improved after stopping therapy^18^.

**Table 2:** Adverse events reported for sofosbuvir-based regimens.

Study	Number of patients	Anemia (%)	Fatigue (%)	Rash or itching (%)	Headache (%)	Nausea/vomiting (%)	Insomnia (%)
Agarwal et al.^14^	62	23 (37%)	NR	NR	NR	NR	NR
Akhil et al.^10^	22	9 (41%)	NR	NR	NR	NR	NR
Choudhary et al.^16^	7	3 (43%)	2	NR	NR	NR	NR
Desnoyer et al.^17^	12	3 (25%)	2 (17%)	1 (8%)	2 (17%)	NR	NR
Gupta et al.^18^	7	1 (14%)	NR	NR	NR	NR	NR
He et al.^19^	33	NR	13 (39%)	NR	NR	13	1
Sperl et al.^11^	6	0	NR	NR	NR	NR	NR
Surendra et al.^22^	21	NR	0	NR	1 (5%)	0	0
Singh T. et al.^21^	8	1 (13%)	NR	1 (13%)	1 (13%)	1	1
Borgia et al.^23^	59	4 (7%)	8	NR	10	8	1
Seo et al.^13^	9	5 (56%)	2	2	NR	NR	1
Lin et al.^27^	7	NR	NR	NR	1	NR	NR
Debnath et al.^25^	18	0 (0%)	2	NR	1	4	NR
Singh A. et al.^29^	39	0 (0%)	NR	1	1	4	4
Mandhwani et al.^28^	73	28 (%)	NR	NR	NR	NR	NR
Gaur et al.^12^	31	2 (38%)	NR	NR	1	NR	NR

*NR* Not reported.

## Discussion

A systematic review and meta-analysis was conducted to determine the safety and efficacy of SOF-based regimens in the treatment of HCV infection among patients requiring maintenance hemodialysis. Among the 934 studies that were identified in our database search, 20 studies met our inclusion criteria. Overall, the pooled estimate of efficacy, based on SVR12, was 95%, ranging from 73 to 100%. Using meta-regression analyses, no statistically significant differences were identified for patient demographics, use of different types of DAA in addition to SOF, or HCV genotypes. Among the studies that reported the dose of SOF, no statistically significant differences in SVR12 were noted with pooled estimates of 92%, 98%, and 100% for SOF doses of 400 mg on alternate days, 400 mg daily and 200 mg daily, respectively.

Among the studies that reported adverse events, anemia was the most common with a pooled prevalence of 15%. A total of 10 (50%) studies reported anemia as an adverse event. Among these, 6 (60%) included ribavirin in the SOF-based regimens. Since ribavirin frequently causes anemia, a subset analysis was performed to compare rates of anemia among ribavirin and ribavirin-free regimens^30^. The prevalence of anemia was significantly higher in regimens containing ribavirin (46%) compared to ribavirin-free regimens (3%). Other adverse events identified in this meta-analysis, included fatigue, rash or itching, headaches, insomnia and nausea or vomiting with pooled estimates of 16%, 7%, 7%,3% and 14%, respectively.

Several large-scale studies evaluated the efficacy and safety among patients with HCV infection and severe renal impairment, treated with SOF-based regimens^8,9^. A longitudinal prospective study by Saxena et al. compared outcomes of HCV infection between 1716 pts with estimated glomerular filtration rate (eGFR) > 45 ml/min/1.73 m^2^ to 73 patients with an eGFR ≤ 45, of which five were on hemodialysis. Patients were treated with SOF 400 mg daily and 60% also received ribavirin. Among patients with eGFR ≤ 45, SVR12 was achieved among 83% of patients. Reported rates of adverse events, in this subset of patients, were similar to our study, with anemia, fatigue and headaches occurring in 30%, 30% and 14% of patients, respectively. Rash or itching was not reported^9^. A meta-analysis by Li et al. evaluated studies of SOF-based regimens among HCV positive patients with stage 4 or 5 chronic kidney disease (GFR < 30 mL/min/1.73 m^2^). Their outcome of efficacy combined SVR at 12 and 24 weeks. Twenty-one studies were included, with a total of 717 HCV infected patients, of whom 306 (58.4%) were on either hemodialysis or peritoneal dialysis. Doses of SOF varied and a subset also received ribavirin. Among patients requiring dialysis, the pooled SVR12/24 was 95%. Rates of anemia, fatigue, headache, rash or itching were not reported^8^. Lastly, in November 2019, the U.S. Food and Drug Administration (FDA) updated the prescribing of several SOF-based regimens, which now states that no dosage adjustment is required for patients with any degree of renal impairment. In the updated labeling report for sofosbuvir/velpatasvir, for example, the FDA reported an overall SVR of 95% among 59 patients requiring hemodialysis and peritoneal dialysis. Adverse events were not provided^31^. Our findings support the notion that the use of Sofosbuvir based regimens in patients receiving hemodialysis is both safe and effective. In our analysis, hemodialysis did not seem to be an unfavorable factor, in terms of safety and efficacy, when compared to other meta-analyses which included Hepatitis C patients without renal disease^32,33^.

There are several limitations that warrant discussion. First, substantial heterogeneity was found among studies, which we addressed by using a random effects model instead of a fixed effects model. Furthermore, meta-regression analysis of specific variables, including patient demographics, HCV genotype and SOF dose did not identify any statistically significant differences. Second, although some publications reported lower SVR rates based on different SOF-based regimens, a subset analyses of types of SOF-based regimens and a comparison of NS5A and NS3 protease inhibitor regimens could not be performed due to the small number of studies in each group. Future studies will need to compare differences in efficacy and safety among SOF-based regimens using different DAA. Third, efficacy by specific HCV genotype could not be determined, again due to the small number of patients and that data pertaining to specific genotypes and outcomes were not extractable.

In conclusion, our findings support that the use of SOF-based regimens in the treatment of HCV infection among patients requiring hemodialysis is both safe and effective. Future randomized controlled studies are needed to confirm these results and to evaluate the optimal SOF-based treatment regimen.

## Methods

This systematic review and meta-analysis was performed in accordance with the Preferred Reporting Items for Systematic Reviews and Meta-Analyses Statement (PRISMA)^34^ and has been registered in PROSPERO (CRD42018116632, https://www.crd.york.ac.uk/PROSPERO).

### Search strategy

A systematic search of Pubmed and Embase databases was performed to identify studies reporting the efficacy and safety of SOF-based regimens for the treatment of HCV positive patients on maintenance hemodialysis. The following search term was used: (end stage renal disease OR renal replacement therapy OR chronic kidney failure OR severe renal impairment OR chronic kidney disease OR hemodialysis) AND (sofosbuvir). Results were limited to the English language and publication dates until May 22, 2020. Additionally, references of systematic reviews focusing on HCV treatment were reviewed manually.

### Eligibility criteria and study selection

Two reviewers (KB and EMCD) independently screened and evaluated the studies that were potentially eligible for inclusion with discrepancies were resolved either between the two reviewers or a third party (FS), if consensus could not be reached. Studies were included if they reported extractable data on the number of maintenance hemodialysis patients that completed a SOF-based regimen and had values for a sustained virologic response 12 weeks after the completion of treatment (SVR12). Our primary outcome of interest was the efficacy of SOF-based therapy in HCV positive patients on maintenance hemodialysis. Regimen efficacy was defined as the proportion of patients that had SVR12. As a secondary outcome of interest, we evaluated the safety of SOF-based regimens, by quantifying the proportion of patients with reported adverse events.

Studies that reported only aggregate data on patients, regardless of their dependency on hemodialysis, were excluded as were opinion pieces and conference abstracts. Studies that focused on patients < 18 years old, HIV positive patients or transplant recipients were also excluded. SOF-based regimens that included pegylated interferon were also excluded.

### Data extraction and quality assessment

For each included study, we extracted the following information: study period, country, type of study, age, gender, HCV genotype, number of patients on a SOF-based therapy, other DAA used in treatment regimen, SOF dose, number of patients who had SVR12, and adverse events. Data extraction was performed by FS and checked for accuracy by EMCD.

We assessed the quality of the eligible studies using the Newcastle–Ottawa Scale^35^. A maximum of six stars could be assigned to each study, as the parameters “selection of the non-exposed cohort” and “comparability between cohorts” were not applicable to our analysis. High quality studies were defined as studies that received ≥ 5 stars (Supplementary Table 1).

### Data synthesis and analysis

We performed a random effects meta-analysis to estimate the pooled efficacy of SOF-based therapy in HCV positive patients on dialysis, using the DerSimonian and Laird approach^36^. The Freeman Tukey double arcsine transformation was used to stabilize the variances^37^. We selected a random effects model, because we assumed that the effects are heterogeneous due to differences in the study design, SOF dosage and combination drugs of each study. Studies were grouped by regimen dosage where possible, and a random effects meta-analysis was carried out in these sub-groups. We assessed the heterogeneity among studies and subgroups using the I^2^ statistic^38^. The Egger’s test was used to explore publication bias and small study effects^39^.

To evaluate the safety of the SOF-based regimens, we performed a random-effects meta-analysis for each adverse event reported in 3 or more studies, and estimated the pooled proportion of patients that reported each adverse event. A meta-regression analysis was also conducted to investigate the extent to which the differences in study characteristics were correlated with the between study heterogeneity^40^. For our review, we used an intention to treat analysis. Patients lost to follow-up were considered as treatment failures.

Stata v15 (Stata Corporation, College Station, TX, USA) was used to perform the statistical analysis. The statistical significance threshold was set at 0.05.

## Supplementary information

Supplementary Information.

## Data Availability

All data generated or analyzed during this study are included in this published article (and its Supplementary Information files).

## References

[1] Nguyen DB, Bixler D, Patel PR (2019). Transmission of hepatitis C virus in the dialysis setting and strategies for its prevention. Semin. Dial..

[2] Jadoul M (2019). Prevalence, incidence, and risk factors for hepatitis C virus infection in hemodialysis patients. Kidney Int..

[3] Polaris Observatory HCVC (2017). Global prevalence and genotype distribution of hepatitis C virus infection in 2015: A modelling study. Lancet Gastroenterol. Hepatol..

[4] Goodkin DA (2017). Mortality, hospitalization, and quality of life among patients with hepatitis C infection on hemodialysis. Clin. J. Am. Soc. Nephrol..

[5] Gordon CE (2019). Prevention, diagnosis, evaluation, and treatment of hepatitis C virus infection in chronic kidney disease: Synopsis of the kidney disease: Improving global outcomes 2018 clinical practice guideline. Ann. Intern. Med..

[6] Jadoul M (2018). Executive summary of the 2018 KDIGO Hepatitis C in CKD Guideline: Welcoming advances in evaluation and management. Kidney Int..

[7] 7Gilead Sciences. Sofosbuvir—Full prescribing information. https://www.accessdata.fda.gov/drugsatfda_docs/label/2015/204671s002lbl.pdf. (2015).

[8] Li M, Chen J, Fang Z, Li Y, Lin Q (2019). Sofosbuvir-based regimen is safe and effective for hepatitis C infected patients with stage 4–5 chronic kidney disease: A systematic review and meta-analysis. Virol. J..

[9] Saxena V (2017). Safety and efficacy of current direct-acting antiviral regimens in kidney and liver transplant recipients with hepatitis C: Results from the HCV-TARGET study. Hepatology.

[10] Akhil MS (2018). Sofosbuvir-based treatment is safe and effective in Indian hepatitis C patients on maintenance haemodialysis: A retrospective study. Nephrology (Carlton).

[11] Sperl J (2017). Combination of sofosbuvir and daclatasvir in the treatment of genotype 3 chronic hepatitis C virus infection in patients on maintenance hemodialysis. Ther. Clin. Risk Manag..

[12] Gaur N (2020). Sofosbuvir–velpatasvir fixed drug combination for the treatment of chronic hepatitis C infection in patients with end-stage renal disease and kidney transplantation. J. Clin. Exp. Hepatol..

[13] Seo HY, Seo MS, Yoon SY, Choi JW, Ko SY (2020). Full-dose sofosbuvir plus low-dose ribavirin for hepatitis C virus genotype 2-infected patients on hemodialysis. Korean J. Intern. Med..

[14] Agarwal SK, Bagchi S, Yadav RK (2017). Hemodialysis patients treated for hepatitis C using a sofosbuvir-based regimen. Kidney Int. Rep..

[15] Bhamidimarri KR (2015). Safety, efficacy and tolerability of half-dose sofosbuvir plus simeprevir in treatment of Hepatitis C in patients with end stage renal disease. J. Hepatol..

[16] Choudhary NS (2017). Efficacy and safety of sofosbuvir-based regimens in chronic hepatitis C patients on dialysis. Indian J. Gastroenterol..

[17] Desnoyer A (2016). Pharmacokinetics, safety and efficacy of a full dose sofosbuvir-based regimen given daily in hemodialysis patients with chronic hepatitis C. J. Hepatol..

[18] Gupta A, Arora P, Jain P (2018). Sofosbuvir based regimen in management of hepatitis C for patients with end stage renal disease on hemodialysis: A single center experience from India. J. Clin. Exp. Hepatol..

[19] He YL (2018). Safety and efficacy of sofosbuvir-based treatment of acute hepatitis C in end-stage renal disease patients undergoing haemodialysis. Aliment Pharmacol. Ther..

[20] Mehta R (2018). Preliminary experience with sofosbuvir-based treatment regimens for patients dependent on hemodialysis. Indian J. Gastroenterol..

[21] Singh T (2016). Sofosbuvir-based treatment is safe and effective in patients with chronic hepatitis C infection and end stage renal disease: A case series. Liver Int..

[22] Surendra M (2018). Ledipasvir and sofosbuvir for untreated HCV genotype 1 infection in end stage renal disease patients: A prospective observational study. Hemodial. Int..

[23] Borgia SM (2019). Sofosbuvir/velpatasvir for 12 weeks in hepatitis C virus-infected patients with end-stage renal disease undergoing dialysis. J. Hepatol..

[24] Cheema SUR, Rehman MS, Hussain G, Cheema SS, Gilani N (2019). Efficacy and tolerability of sofosbuvir and daclatasvir for treatment of hepatitis C genotype 1 & 3 in patients undergoing hemodialysis: A prospective interventional clinical trial. BMC Nephrol..

[25] Debnath P (2020). Combined Ns5a & Ns5b nucleotide inhibitor therapy for patients with chronic hepatitis C with stage 5 chronic kidney disease on hemodialysis. Arq. Gastroenterol..

[26] Hussein NR, Saleema ZSM, Abd QH (2019). Direct acting antiviral treatment for patients with end-stage kidney disease with acute HCV infection. Mediterr. J. Hematol. Infect. Dis..

[27] Lin T (2020). Effect of hemodialysis on efficacy and pharmacokinetics of sofosbuvir coformulated with either daclatasvir or ledipasvir in patients with end-stage renal disease. Blood Purif..

[28] Mandhwani R (2020). Use of sofosbuvir based regimen in patients with end-stage renal disease and chronic hepatitis C; an open label, non-randomized, single arm, single center study from Pakistan. Gastroenterol. Hepatol. Bed Bench.

[29] Singh A, Kumari S, Kumar P, De A, Singh V (2018). Sofosbuvir with NS5A inhibitors in hepatitis C virus infection with severe renal insufficiency. J. Viral. Hepat..

[30] Canonico PG (1984). Effects of ribavirin on red blood cells. Toxicol. Appl. Pharmacol..

[31] Gilead Sciences. EPCLUSA(sofosbuvir and velpatasvir) tablets, for oral use: Prescribing information. https://www.gilead.com/-/media/files/pdfs/medicines/liver-disease/epclusa/epclusa_pi.pdf. (2019).

[32] Dolatimehr F (2017). Combination of sofosbuvir, pegylated-interferon and ribavirin for treatment of hepatitis C virus genotype 1 infection: A systematic review and meta-analysis. Daru.

[33] Yang HJ, Ryoo JY, Yoo BK (2015). Meta-analysis of the efficacy and safety of sofosbuvir for the treatment of hepatitis C virus infection. Int. J. Clin. Pharm..

[34] Moher, D., Liberati, A., Tetzlaff, J., Altman, D. G. & Group, P (2009). Preferred reporting items for systematic reviews and meta-analyses: The PRISMA statement. PLoS Med..

[35] Wells G (2016). The Newcastle-Ottawa Scale (NOS) for Assessing the Quality of Nonrandomised Studies in Meta-analyses.

[36] DerSimonian R, Laird N (1986). Meta-analysis in clinical trials. Control Clin. Trials.

[37] Nyaga VN, Arbyn M, Aerts M (2014). Metaprop: A Stata command to perform meta-analysis of binomial data. Arch. Public Health.

[38] Higgins JP, Thompson SG (2002). Quantifying heterogeneity in a meta-analysis. Stat. Med..

[39] Peters JL, Sutton AJ, Jones DR, Abrams KR, Rushton L (2006). Comparison of two methods to detect publication bias in meta-analysis. JAMA.

[40] Harbord RM, Higgins JPT (2008). Meta-regression in Stata. Stata J..

