# Supplementary material for: Efficacy and safety of sofosbuvir in the treatment of hep C among patients on hemodialysis: a systematic review and meta-analysis
Source: Sci Rep. 2020 Aug 31;10:14332. doi: 10.1038/s41598-020-71205-5 (PMC7459301; doi:10.1038/s41598-020-71205-5)

Efficacy and safety of sofosbuvir in the treatment of hep C among patients on hemodialysis: a systematic review and meta-analysis

Supplementary Information

Fadi Shehadeh MEng[1*], Markos Kalligeros MD[1], Katrina Byrd MD[1], Douglas Shemin[2] MD, Eleftherios Mylonakis[1] MD PhD., Paul Martin MD[3], Erika M.C D'Agata MD MPH[1]

[1] Infectious Diseases Division, Warren Alpert Medical School of Brown University, Rhode Island Hospital, Providence, RI, [2] Kidney Disease Division, Warren Alpert Medical School of Brown University, Rhode Island Hospital, Providence, RI, [3] Division of Digestive Health and Liver Disease, Miller School of Medicine, University of Miami, Miami FL.

Supplementary Table 1 - Newcastle-Ottawa Scale (NOS)								
	SELECTION				COMPARABILITY	OUTCOME		
	Representativeness of the exposed cohort	Selection of the non-exposed cohort	Ascertainment of exposure	Demonstration that outcome of interest was not present at start of study	Comparability of cohorts on the basis of the design or analysis	Assessment of outcome	Follow-up long enough for outcomes to occur	Adequacy of follow-up of cohorts
Agarwal et al., 2017	★	NA	★	★	NA	★	★	★
Akhil et al., 2017	★	NA	★	★	NA	★	★	★
Bhamidimarri et al., 2015	★	NA	★	★	NA	★	★	★
Choudhary et al., 2017	★	NA	★	★	NA	★	★	★
Desnoyer et al., 2016	★	NA	★	★	NA	★	★	★
Gupta et al., 2018	★	NA	★	★	NA	★	★	★
He et al., 2017	★	NA	★	★	NA	★	★	★
Mehta et al., 2018	★	NA	★	★	NA	★	★	★
Spertl et al., 2017	★	NA	★	★	NA	★	★	★
Surendra et al., 2018	★	NA	★	★	NA	★	★	★
Singh T. et al., 2016	★	NA	★	★	NA	★	★	★
Borgia et al., 2019	★	NA	★	★	NA	★	★	★
Seo et al., 2020	★	NA	★	★	NA	★	★	★
Lin et al., 2019	★	NA	★	★	NA	★	★	★
Debnath et al., 2019	★	NA	★	★	NA	★	★	★
Singh A. et al., 2018	★	NA	★	★	NA	★	★	★
Mandhwani et al., 2020	★	NA	★	★	NA	★	★	★
Hussein et al., 2019	★	NA	★	★	NA	★	★	★
Gaur et al., 2020	★	NA	★	★	NA	★	★	★
Cheema et al., 2019	★	NA	★	★	NA	★	★	★

Supplementary Figure 1 - Individual and combined estimates of the efficacy of sofosbuvir -based therapy in patients with cirrhosis with 95% confidence intervals. ES: Effect Size (Efficacy)

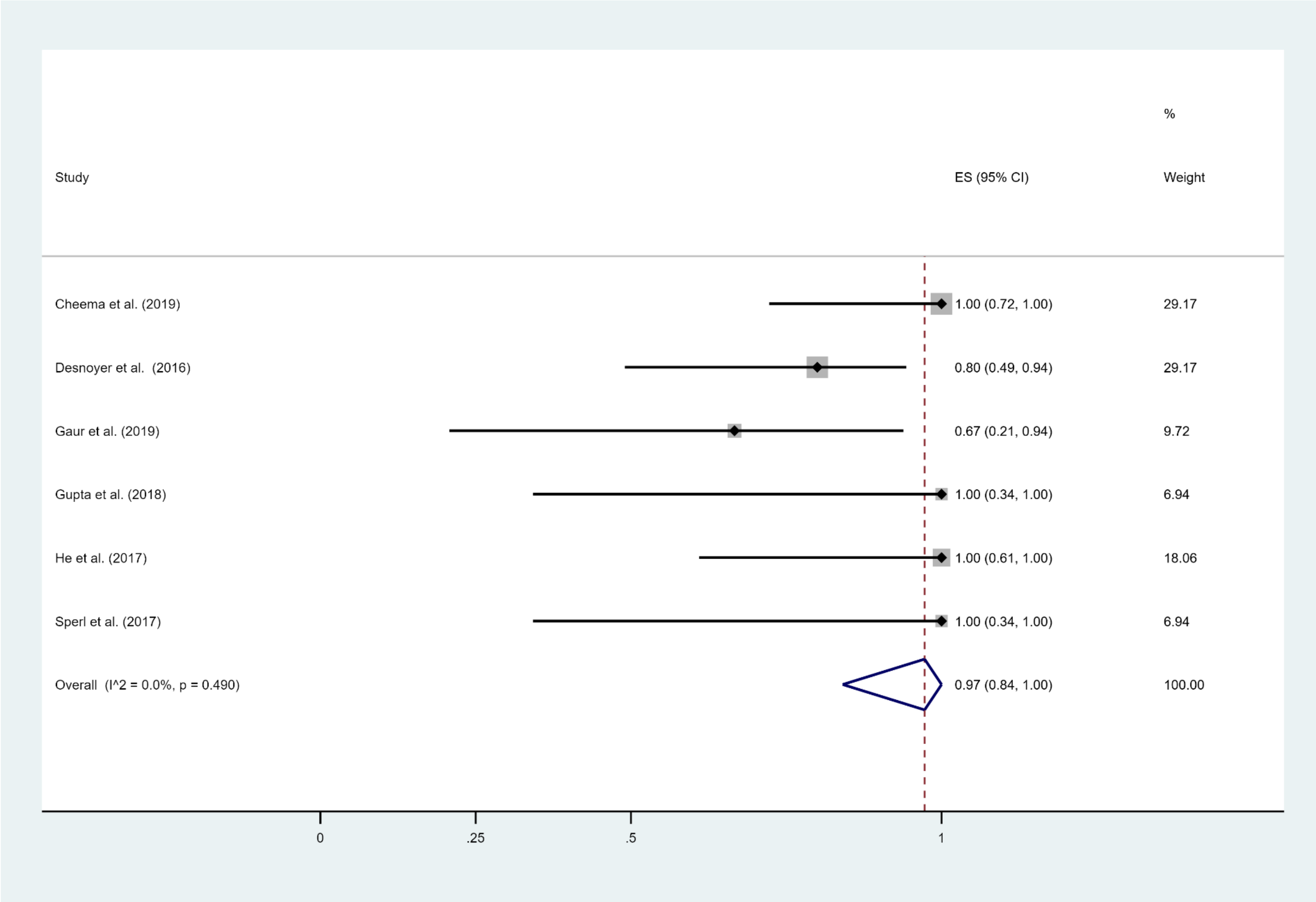

Supplement: Supplementary file 1 — Supplementary Information. [file 41598_2020_71205_MOESM1_ESM.pdf]
